# Evaluating pediatrics residents’ ethics learning needs using multisource interprofessional feedback

**Published:** 2017-12-15

**Authors:** Peter MacPherson, Julie Emberley

**Affiliations:** 1 Department of Pediatrics, School of Medicine, Queen’s University, Kingston General Hospital, Ontario, Canada; 2 Faculty of Medicine, Memorial University of Newfoundland, Newfoundland, Canada

## Abstract

**Background:**

Ethics education is a required component of pediatric residency training. Limited instructional time requires educators to identify and prioritize learning needs. This is the first study to identify pediatric residents’ ethics learning needs using a multisource (360 degree) assessment. We hypothesized that pediatricians or allied health care professionals would identify unperceived ethics learning needs.

**Methods:**

Pediatric residents, pediatricians, respiratory therapists (RTs), and registered nurses (RNs) working at a university children’s hospital rated the importance of twelve ethics themes as learning needs for trainees using a Likert-type scale. One-way ANOVA was used to determine differences between the groups, followed by post-hoc testing.

**Results:**

Response rates were 65%, 70%, 57%, and 47% for residents, pediatricians, RTs, and RNs, respectively. Themes were categorized into three priority groupings based on mean importance ratings. Where significant differences existed between residents and other respondent groups, pediatric residents rated the theme as being more important.

**Conclusion:**

This study provides an interprofessional assessment of pediatric residents’ perceived ethics learning needs. High priority ethics topics were identified, allowing for targeted teaching. Pediatricians and allied HCPs did not rate any ethics themes higher than residents. Medical educators may consider using methods inspired by multisource feedback for program evaluation.

## Introduction

Ethics and professionalism education is a required component of pediatric residency training in North America.^1^,^2^ However, most pediatric residency programs lack structured ethics and professionalism curricula.^3^,^4^ Among the published ethics curricula for pediatric residents,^5^–^8^ no consensus exists as to what topics should be included. Ethics is generally taught on an *ad hoc* basis, with fewer than eleven instructional hours allocated to ethics topics annually.^4^ Pediatric program directors identify curricular crowding as the main barrier to ethics education.^9^ A recent review on pediatric residents’ ethics education concluded that current training was generally not adequate.^10^

Expert opinion, as reflected in published curricula, has an important role to play in guiding ethics and professionalism education. However, educational needs assessments are a crucial adjunct to expert opinion in order to determine trainees’ learning needs, particularly in the setting of limited instructional time.

Multisource feedback (360 degree assessment) is predominantly used in the assessment of clinical competence. In this study, we apply this strategy to the world of program evaluation to consider educational needs from multiple perspectives.

The primary goal of this study was to identify and prioritize pediatric residents’ ethics learning needs using an interprofessional assessment that incorporated the perceptions of pediatricians and allied health care professionals (HCPs). This is novel as previous studies within pediatrics have been limited to trainees’ self-reported needs alone.^11^,^12^ Our secondary goal was to determine if differences exist between residents’ ethics learning needs as perceived by residents, pediatricians and allied HCPs. Previous research found that nurses identified professional interactions with patients and colleagues as an unperceived learning need for emergency medicine residents.^13^ We hypothesized that pediatricians or allied HCPs would identify learning needs not perceived by pediatric residents.

## Methods

This study took place at a university children’s hospital in Canada that has a pediatric residency training program. Ethics and professionalism teaching in the program was largely *ad hoc*, with the exception of several academic half-day sessions delivered to all first-year medical residents at our university. These sessions were not targeted towards pediatric trainees. Eligible participants were pediatric residents (*N*=23), general and subspecialty pediatricians (*N*=43), respiratory therapists (RTs; *N*=14), and registered nurses (RNs; *N*=151) working at this institution. Residents from all four postgraduate years participated. RNs and RTs working in the emergency department, pediatric ward, and intensive care units were sampled. RNs and RTs were selected because they work closely with residents on cases with ethical dimensions (e.g., premature infants at the limits of viability) and interact in non-supervisory roles. Ethics approval was obtained from the provincial Health Research Ethics Authority.

Participants completed a survey regarding the importance of twelve ethics themes as learning needs for pediatric residents (see eSupplement) similar to the methodology employed by Pauls and Ackroyd-Stolarz.^12^ Ethics themes were distilled from a review of published ethics curricula for pediatric residents.^5^–^8^ Three key informants reviewed the list of ethics themes for completeness and content validity. The informants were a pediatric bioethicist, a pediatric subspecialist with postgraduate training in ethics (*Senior author’s initials, removed for blind review*), and a general pediatrician who recently graduated from the residency program. No new themes were identified by the key informants.

Participants were asked to rate the importance of each ethics theme as a learning need for trainees using a five point Likert-type scale. The scale ranged from a score of 1 (Not important) to a score of 5 (Very important). Residents were asked about the importance and adequacy of their ethics education. Paper surveys were distributed to residents, RNs, and RTs in clinical areas. The survey was distributed to pediatricians using the FluidSurveys online platform.

A rank list was generated for each group by arranging mean importance scores in descending order. Each theme’s rank was averaged between the four groups. This average ranking was used to generate an interprofessional ranking of the themes’ relative importance. The themes were separated into three priority groupings (high, intermediate, and low) with the aim of prioritizing teaching sessions.

Results were analyzed using Stata 11 (Statacorp, College Station, TX). Importance on the Likert-type scale was considered as an interval variable.^14^,^15^ One-way ANOVA tests were used to assess whether there were significant differences between the groups’ mean importance ratings for each ethics theme. Bonferroni post-hoc tests were used for pairwise comparisons where significant between-group differences were found.

## Results

Response rates were 65%, 70%, 57%, and 47% for residents (*n*=15), pediatricians (*n*=30), RTs (*n*=8), and RNs (*n*=71), respectively. Residents from all years of training participated (7 junior residents, 8 senior residents).

The majority of residents (73%, 11 of 15) felt that ethics education was very important to their overall education and 53% (8 of 15) of residents felt that their ethics education was less than adequate.

All four respondent groups rated ethics themes as important learning needs for pediatric residents with mean importance scores ranging from 3.1 to 4.9 (where 3 was ‘Somewhat important,’ 4 was ‘Important,’ and 5 was ‘Very important’). Results are shown in Table 1.

The twelve themes were divided into three priority groupings (high, intermediate, and low) on the basis of the average numerical rank of each theme (Table 2). The high priority themes identified were: ethics of death and dying/withdrawal and withholding of life-sustaining treatment; ethical issues in the neonatal intensive care unit (NICU); truth-telling, confidentiality and disclosure of error; and informed consent and capacity in pediatrics.

The mean importance differed significantly between the groups for seven ethics themes, but post-hoc testing revealed significant pairwise differences in only six instances (Table 1). Of these six instances, residents rated genetic testing and screening as significantly more important than RTs or RNs. In the other five instances, residents did not differ significantly from the other respondent groups (e.g., in most cases, the significant pairwise difference observed was between pediatricians and RTs/RNs and not relevant to our hypothesis).

## Discussion

Consistent with previous research,^12^ ethics themes were recognized as important learning needs for residents in our study. Most residents described their ethics education to date as less than adequate. Similarly, prior studies found that many practicing pediatricians rate the ethics education they received during residency as poor or fair^16^–^18^ and that knowledge of ethics once in practice was lacking in several important domains.^19^

Recognizing the significant challenge of curricular crowding, the higher and intermediate priority ethics themes presented in Table 2 could be preferentially addressed in a targeted fashion by implementing and/or adapting existing curricula. Given the generally high importance ratings in the present study, caution should be exercised in excluding topics altogether. We separated themes into three priority groups on the basis of mean importance ranks. However, the themes included in the low priority group generally had mean importance ratings between 3 (‘Somewhat important’) and 4 (‘Important’).

Contrary to our hypothesis, pediatricians and/or allied HCPs did not rate any ethics themes as more important than did residents. This suggests that pediatric residents in our center did not have unperceived ethics learning needs. In contrast, a similar study found that nurses identified a learning need (communication) not perceived by emergency medicine residents.^13^ This may represent a difference between medical specialties, institutional contexts or topic areas.

This study has several limitations. It was conducted at a single center and therefore our results may not be generalizable. However, it is hoped that interested readers can gauge the applicability to their settings by virtue of the description of our institutional context. We examined perceived ethics learning needs and it is possible that unperceived needs went unrecognized. However, inclusion of other professionals’ perceptions should have helped to minimize this possibility. Future needs assessments might consider use of objectives measures, such as objective structured clinical examination (OSCE) stations exploring ethics themes, in addition to the use of perceived needs.

The instrumentation used in this study has practical advantages that could also be construed as a threat to validity. The twelve ethics themes included in the survey were distilled from modules/topics in published ethics curricula such that each theme could constitute a separate learning item (e.g., an academic half-day session or online learning module). Practically, this gives clinician educators a framework with which to organize ethics teaching in their institution. However, a manageable number of topics sometimes required grouping two or more related constructs into one survey item (e.g., research ethics and ethics of innovation). In so doing, we made the pragmatic decision to use twelve broad themes rather than a multitude of conceptually distinct survey items.

In conclusion, this study provides an interprofessional assessment of pediatric residents’ perceived ethics learning needs at our center. Expert opinion and competency frameworks have an important role to play in guiding ethics and professionalism education, but require application and interpretation by clinician educators in particular contexts. The present study compliments the existing literature on ethics education in pediatric residency training by identifying high priority ethics topics, which may mean de-emphasizing lower priority topics in the context of limited instructional time. However, consideration should also be given to increasing curricular time allocated in residency training to ethics and professionalism education at the expense of sessions centered on the Medical Expert CanMEDS role.

## Supplementary Information



## Figures and Tables

**Table 1:** Importance of ethics themes as learning needs for pediatric residents. The importance of each theme was rated by four groups of respondents from 1 to 5 on a Likert-type scale, where 1 was ‘Not important,’ 3 was ‘Somewhat important,’ and 5 was ‘Very important’. The mean importance was calculated for each group of respondents. Significance was assessed as *p*<0.05 using one-way ANOVA tests. NS, not significantly different. Post-hoc testing revealed pairwise differences between the respondent groups in six instances. Only one of these instances was relevant to the study’s hypothesis: residents rated genetic testing and screening higher than did RNs or RTs

Ethics Theme	Importance of Ethics Theme as a Learning Need Mean (SD)				
	Pediatric Residents (*n*=15)	Pediatricians (*n*=30)	Respiratory Therapists (*n*=8)	Registered Nurses (*n*=71)	F statistic
Ethics of death and dying/withdrawal and withholding of life-sustaining treatment	4.6 (0.5)	4.6 (0.5)	4.8 (0.5)	4.8 (0.5)	NS
Ethical issues in the neonatal intensive care unit (NICU)	4.6 (0.6)	4.5 (0.6)	4.6 (0.5)	4.8 (0.5)	NS
Truth-telling, confidentiality and disclosure of error	4.6 (0.5)	4.9 (0.3)	4.3 (0.9)	4.7 (0.5)	F (3,120) = 3.49, *p*<0.05
Informed consent and capacity in pediatrics	4.6 (0.5)	4.6 (0.8)	3.8 (0.9)	4.4 (0.8)	F (3,119) = 3.06, *p*<0.05
Ethical decision-making in the context of family-centered care	4.5 (0.6)	4.6 (0.6)	4.3 (0.5)	4.4 (0.7)	NS
Religious, cultural and philosophical objections to care	4.3 (0.6)	4.6 (0.6)	3.9 (0.4)	4.2 (0.8)	F(3,120) = 3.39, *p*< 0.05
Residency training issues	4.4 (0.7)	4.4 (0.7)	3.8 (0.7)	3.8 (0.9)	F(3,118) = 5.63, *p*<0.01
Resource allocation/ justice and social responsibility	4.1 (0.7)	3.9 (0.9)	3.8 (1.2)	4.0 (1.0)	NS
Research ethics and ethics of innovation	3.8 (0.6)	4.2 (0.5)	3.5 (0.5)	3.5 (0.9)	F(3,120)= 4.70, *p*<0.01
Genetic screening and testing	4.4 (0.7)	4.0 (0.7)	3.1 (1.1)	3.5 (1.0)	F(3,120) = 6.21, *p*<0.001
Conflicts of interest and professionalism	3.9 (0.9)	4.2 (0.7)	3.1 (0.8)	3.4 (0.9)	F(3,118) = 7.12, *p*<0.001
Transplantation	3.7 (0.6)	3.7 (0.9)	3.6 (1.1)	3.9 (0.8)	NS

**Table 2:** Relative importance of ethics themes as learning needs for pediatric residents. The mean importance ratings from Table 1 were used to generate a rank list of ethics themes for each group of respondents, with 1 being the most important and 12 being the least important. An average rank for the four groups was obtained by averaging the rating given by each group. The twelve themes were separated into three priority groupings.

Ethics Theme	Rank of Ethics Theme as a Learning Need				
	Pediatric Residents	Pediatricians	Respiratory Therapists	Registered Nurses	Average Rank
**High Priority**					
Ethics of death and dying/withdrawal and withholding of life-sustaining treatment	1*	5	1	1*	2
Ethical issues in the neonatal intensive care unit (NICU)	1*	6	2	1*	2.5
Truth-telling, confidentiality and disclosure of error	4	1	3*	3	2.8
Informed consent and capacity in pediatrics	1*	2*	6	5	3.5
**Intermediate Priority**					
Ethical decision-making in the context of family-centered care	5	4	3*	4	4
Religious, cultural and philosophical objections to care	6	2*	5	6	4.8
Residency training issues	7*	7	7*	9	7.5
Resource allocation/ justice and social responsibility	9	11	7*	7	8.5
**Low Priority**					
Research ethics and ethics of innovation	11	8*	10	10	9.8
Genetic screening and testing	7*	10	11*	12	10
Conflicts of interest and professionalism	10	8*	11*	12	10.3
Transplantation	12	12	9	8	10.3

* denotes a numerical tie between two or three themes within one respondent group.
