# Supplementary material for: Evaluating pediatrics residents’ ethics learning needs using multisource interprofessional feedback
Source: Can Med Educ J. 2017 Dec 15;8(4):e86–91. (PMC5766223)
Supplement: Supplementary file 1 [file CMEJ-08-86-s001.pdf]

Appendix A. Survey instrument

There are a number of different topics related to ethics that are often included in ethics teaching for paediatric residents. Please rate the importance of each topic as a learning need for paediatric residents on a scale of 1 to 5. **A rating of 1 represents a topic of no importance** to residents and that should not be included within the ethics curriculum. **A rating of 5 represents a topic that you feel is critically important** to the ethics education of residents.

	**1 Not Important**	**2 Of Little Importance**	**3 Somewhat Important**	**4 Important**	**5 Very Important**
**1. Truth-telling, Confidentiality and Disclosure of Error** e.g. Are there times when it is acceptable *not* to tell a patient the truth? What are the limits to physician-patient confidentiality? What is an adverse event and when/how should such events be disclosed to patients and families?					
**2. Informed Consent and Capacity In Pediatrics** e.g. What is required for informed consent? When is a child or adolescent considered capable of making his or her own treatment decisions?					
**4. Ethical Issues in the Neonatal Intensive Care Unit (NICU)** e.g. What are the limits of viability? How do physicians counsel parents at the limits of viability?					
**6. Religious, Cultural and Philosophical Objections to Care** e.g. Can a patient or parents of Jehovah's witness faith refuse a life-saving blood transfusion? What happens if a parent chooses complementary alternative health care treatment for a malignancy instead of chemotherapy?					
**7. Conflicts of Interest and Professionalism** e.g. Is it acceptable for physicians to receive gifts from pharmaceutical companies? Should pediatricians Facebook friend their patients?					

	1<br>Not Important	2<br>Of Little Importance	3<br>Somewhat Important	4<br>Important	5<br>Very Important
**9. Research Ethics and Ethics of Innovation**<br>e.g. Are pediatric patients in need of special protections in research studies? How do we balance the development of new technologies and patient safety?					
**10. Transplantation**<br>e.g. Should adolescent be able to be a living related kidney donor for his/her parent? What are the ethical implications of using IVF to create a potential bone marrow donor for a sibling?					
**12. Residency Training Issues**<br>e.g. How should a resident respond to unprofessional behavior by a supervisor or colleague? Should residents disclose to families that it is their first time performing a procedure?					

[The following questions were posed to pediatric residents only]

I feel that the ethics education I have received to date during my paediatric residency training is:

1. Less than adequate
2. Adequate
3. More than adequate

I believe that ethics education is:

1. Not important to my residency training
2. Somewhat important to my residency training
3. Very important to my residency training
